# Packed red blood cell transfusion trigger in the intensive care unit of a university hospital

**DOI:** 10.5935/0103-507X.20200024

**Published:** 2020

**Authors:** Maria Paulina Viana Miquilino, Carlos Eduardo Cardoso, Victória Castello Branco Fernandes Martins, Sandra Mara Silva de Almeida, Aparecida Carmem de Oliveira, Gerson Luiz de Macedo

**Affiliations:** 1 Hospital Universitário de Vassouras, Universidade de Vassouras - Vassouras (RJ), Brazil.

To the Editor,

Anemia is a pathological condition with high prevalence and great importance in intensive care units (ICUs) because it increases mortality in critically ill patients. For this reason, the transfusion of packed red blood cells (PRBCs) is a routinely adopted practice, but the safe pre-transfusion hemoglobin (Hb) value indicative of transfusion is still discussed because, although the anemic condition increases the risk of death, blood transfusion is not without risk.^(1-3)^ Recent studies comparing a restrictive transfusion strategy (Hb < 7g/dL) with a more liberal transfusion strategy (Hb < 10g/dL) concluded that, except in surgical patients and in patients with cardiac conditions, the restrictive strategy is possibly superior to the liberal strategy.^(4,5)^

To determine the transfusion trigger for PRBCs at *Hospital Universitário de Vassouras* (HUV), an analysis was conducted of the HUV Hemotherapy Center’s database of transfusions performed from January 2014 to July 2017.

**Figure 1:**
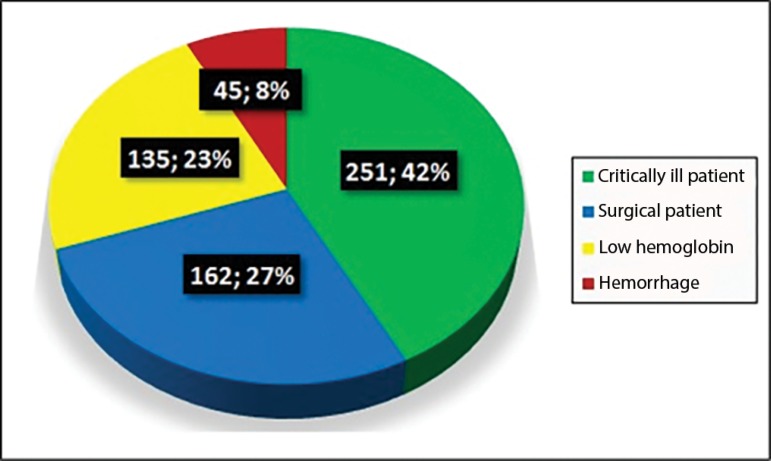
Indications for packed red blood cell transfusion in the intensive care unit.

Of the 611 patients who received transfusions in the ICU, 326 were male (53.4%), and the mean age of the sample was 59.9 years. The transfusion trigger for PRBCs at the HUV ICU was 6.9g/dL. The mean number of PRBC bags used was 1.9 units per patient. For better evaluation of the results, indications for PRBC transfusion were classified into four groups, as shown in figure 1: surgical patient (preoperative and postoperative), hemorrhage, critically ill patient (nonsurgical and nonhemorrhagic), and low Hb. The main indications were low Hb (22.1%), cardiac surgery (10.5%) and sepsis/septic shock (8.7%). The mean pretransfusion Hb level in patients undergoing cardiac surgery was 7.8g/dL.

The mean pretransfusion Hb level found (6.9g/dL) was lower than that reported in other international studies but is similar to the value found in studies in Brazilian ICUs. A recent retrospective study involving 730 ICUs from 84 countries reported a mean pretransfusion Hb value of 8.3g/dL, ranging from 7.8g/dL in Middle Eastern countries to 8.9g/dL in Eastern Europe.^(3)^ Regarding the available data on Brazilian ICUs, a prospective multicenter study conducted in 19 ICUs found a transfusion trigger of 7.7g/dL,^(1)^ while another prospective study conducted in five university hospital ICUs revealed a pretransfusion Hb value of 6.9g/dL.^(6)^ Since the end of the 1990s, restrictive transfusion strategies in critically ill patients have gained greater credibility because they are associated with a lower mortality rate compared with that for more liberal strategies, with the exception of some patients, such as those with perioperative conditions.^(2,4,5,7)^ Surgical patients have an oxygen demand that differs from that of critically ill patients because they are exposed to different conditions, such as surgery-related blood loss, vascular changes due to the effect of anesthesia and fluid changes.^(7)^ In its PRBC transfusion guidelines published in 2016, the American Association of Blood Banks (AABB) recommends a restrictive strategy (Hb <7g / dL) for critically ill non-surgical patients and suggests that red blood cell transfusion is based on Hb level, in the clinical context and patient preferences, and that alternative therapies are considered.^(8)^

Of the main transfusion indications, cardiac surgery was the second most prevalent at HUV. The transfusion trigger of patients undergoing cardiac surgery was 7.8g/dL. Although the literature advocates the liberal strategy for PRBC transfusion in surgical patients, patients undergoing cardiac surgery deserve special attention. Some studies were inconclusive regarding the best transfusion strategy recommended for patients with cardiac comorbidities or undergoing cardiac surgery.^(2,9)^ However, a recent meta-analysis involving 8,886 patients undergoing cardiac surgery showed that the restrictive strategy is as effective and safe as the liberal strategy, as no differences in 30-day mortality or in the morbidity of patients receiving either strategy were observed.^(10)^

To reduce the morbidity and mortality of critically ill patients related to transfusion practices, the pretransfusion Hb value is of great clinical relevance. In the present study, the PRBC transfusion trigger in the HUV ICU was 6.9g/dL. This value follows the restrictive standard recommended by the literature to reduce morbidity and mortality in critically ill patients.
